# Is lean mass quantity or quality the determinant of maximal fat oxidation capacity? The potential mediating role of cardiorespiratory fitness

**DOI:** 10.1080/15502783.2025.2455011

**Published:** 2025-01-29

**Authors:** Edgardo Opazo-Díaz, Juan Corral-Pérez, Alejandro Pérez-Bey, Alberto Marín-Galindo, Adrián Montes-de-Oca-García, María Rebollo-Ramos, Daniel Velázquez-Díaz, Cristina Casals, Jesús-Gustavo Ponce-González

**Affiliations:** a University of Cadiz, ExPhy Research Group, Department of Physical Education, Puerto Real, Spain; b Instituto de Investigación e Innovación Biomédica de Cádiz (INiBICA), Cadiz, Spain; c University of Chile, Exercise Physiology Lab, Physical Therapy Department, Santiago, Chile; d University of Cadiz, GALENO Research Group, Department of Physical Education, Faculty of Education Sciences, Cadiz, Spain; e Neuroscience Institute, Advent Health Research Institute, Orlando, FL, USA

**Keywords:** Lipid metabolism, hand strength, VO_2_max, body composition, metabolic flexibility, muscle mass

## Abstract

**Background:**

Impaired fat oxidation is linked to cardiometabolic risk. Maximal fat oxidation rate (MFO) reflects metabolic flexibility and is influenced by lean mass, muscle strength, muscle quality – defined as the ratio of strength to mass – and cardiorespiratory fitness. The relationship between these factors and fat oxidation is not fully understood. The aim is to analyze the associations of lean-mass, muscle strength and quality with fat oxidation parameters in young adults, considering the mediating role of VO_2_max.

**Methods:**

A cross-sectional observational study. Eighty-one adults (50 males, 31 females; age 22.8 ± 4.4, BMI 25.70 ± 5.75, lean-mass 54.19 ± 8.78, fat-mass 18.66 ± 11.32) Body composition assessment by bioimpedance determine fat and lean-mass. Indirect calorimetry at rest and exercise was used for the calculation of fat oxidation. An incremental exercise protocol in a cycle ergometer with two consecutive phases was performed. The first to determine MFO consisted of 3 min steps of 15W increments with a cadence of 60rpm. The test was stopped when RQ ≥ 1. After 5 min rest, a phase to detect VO_2_max began with steps of 15W/min until exhaustion. Muscular strength was assessed by handgrip dynamometry and the standing longitudinal jump test. A strength cluster was calculated with handgrip and long jump adjusted by sex and age. Data were analyzed using multiple linear regression and mediation analyses.

**Results:**

Total lean-mass and leg lean-mass were not associated with MFO. Long jump, relativized by lean-mass and by leg lean-mass have a standardized indirect effect on MFO of 0.50, CI: 0.32–0.70, on MFO/lean-mass 0.43, CI:0.27–0.60 and MFO/leg lean-mass 0.44, CI: 0.30–0.06, which VO_2_max mediated, VO_2_max/lean-mass and VO_2_max/leg lean-mass, respectively (all *p* < 0.01). The handgrip/arm lean-mass had an indirect effect of 0.25 (CI: 0.12–0.38) on MFO/leg lean-mass, with VO_2_max/leg lean-mass as the mediator (*p* < 0.01). The Cluster/lean-mass and Cluster/Extremities lean-mass have a standardized indirect effect on MFO/lean-mass (0.34, CI: 0.20–0.48) and MFO/leg lean-mass (0.44, CI: 0.28–0.60), mediated by VO2max/lean-mass and VO2max/leg lean-mass (*p* < 0.01).

**Conclusions:**

Muscular strength and quality have an indirect effect on MFO mediated by VO_2_max. These findings suggest the importance of muscle quality on MFO.

## 1. Introduction

An impaired capacity to oxidize fat is associated with increased cardiometabolic risk and reduced overall health [1]. A low reliance on lipids for energy metabolism can result in the accumulation of intramuscular lipids, which may disrupt critical physiological functions [2]. The highest rate of fat oxidation during incremental exercise, known as maximal fat oxidation (MFO), and the corresponding exercise intensity at which it occurs related to maximum oxygen uptake (VO_2_max), referred to as Fatmax, are key markers of metabolic flexibility [3]. Many factors have been identified as determinants of MFO during exercise [4]. While some of these factors, such as age and sex, are non-modifiable [5], others related to physical fitness, like training status, can be modified [6].

Body composition, cardiorespiratory fitness (CRF), and muscular strength are physical fitness components related to health. In particular, CRF, determined as VO_2_max, has been identified as the main determinant of MFO [7]. Additionally, body composition variables – such as body mass, body mass index (BMI), fat mass, and lean mass – have been shown to correlate with MFO and Fatmax significantly [7,8]. Interestingly, previous studies have indicated that there are no significant differences in MFO, even when adjusted for fat-free mass, between individuals with high or low body fat (defined as more or less than 25% body fat), provided that they are matched by VO_2_max [9]. This suggests that lean mass may play an important role in exercise-related fat oxidation [10].

Muscle strength has been shown to have a strong relationship with lean mass [11], and both factors appear to play a role in fat oxidation (Kirk et al., 2009). Moreover, factors influencing MFO are often linked to VO_2_max, a key determinant of metabolic flexibility [4,7]. Importantly, research suggests that lean mass may impact MFO independently of fat mass and VO_2_max, further underscoring its critical role in exercise metabolism (Randell et al., 2017).

According to a review article, individuals with obesity tend to exhibit higher MFO when VO_2_max is considered [7]. However, after adjusting for lean mass, individuals with leaner bodies show higher MFO per unit of metabolically active tissue, regardless of VO_2_max [12]. This highlights the importance of lean mass in fat oxidation. Despite this, the role of muscular strength is often overlooked in studies on fat oxidation, either as an independent factor or as a mediator. Given the close relationship between lean mass and MFO, it is essential to thoroughly explore the connection between muscle strength and MFO. Muscle quality, defined as the ratio of muscle strength and lean mass [13], could provide novel insights into fat oxidation, because the analysis of the functional activity per unit of mass. Understanding this relationship presents a challenge in studying metabolic flexibility, as both lean mass and muscle strength may influence MFO in distinct but interconnected ways.

Therefore, we aimed to analyze the associations of lean mass, muscle strength and quality with MFO and Fatmax in young adults, while also considering the mediating role of VO_2_max.

## 2. Method

### 2.1. Sample

Participants were recruited from the University of Cádiz and were part of the Study of nutritional habits and physical activity level in adults (NUTAF project). The inclusion criteria for this study required participants to be between 18 and 45 years old, to have a BMI within the range of 18.5 to 40 kg/m^2^, and to have a stable body weight in the last 6 months. Also, participants who were smokers, suffered from cardiovascular or metabolic disease or had an injury that did not allow them to exercise were excluded. Participants were not controlled for exercise habits or specific diets. The sample comprises 81 participants, 50 men and 31 women, aged 22.8 ± 4.4 years old.

#### 2.1.1. Procedure

This is an observational cross-sectional study. Following the Helsinki Declaration, the study was approved by the “Puerta del Mar Hospital Ethics Committee” from Cádiz (Spain). Written informed consent was obtained from all participants after they were informed of the nature, protocols, and mean risks. Data collection took place from January 2016 to June 2017.

**Figure 1.**
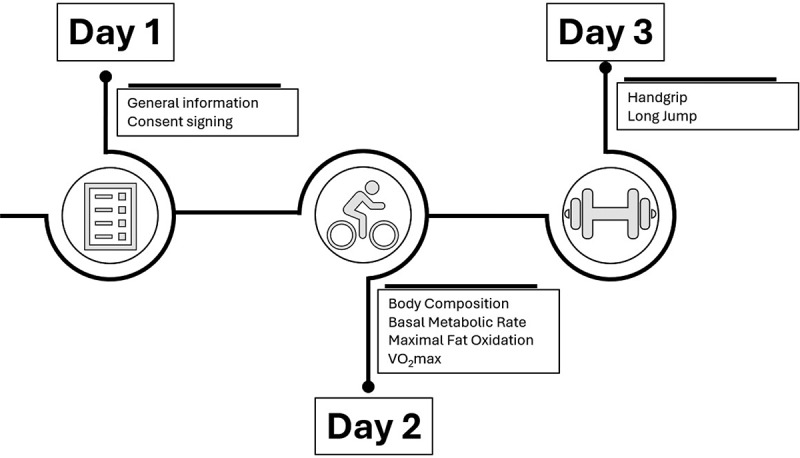
Schematic representation of the study design. VO_2_max: maximum oxygen uptake.

The parameters of body composition, basal metabolism, MFO, and VO_2_max were measured in a single day in the morning (from 8:30 to 11:30am) to maintain a fasting state of at least 8 h and preclude possible circadian rhythm effects. Other previous considerations that were followed 24 h before assessments were to refrain from vigorous exercise and not to take alcoholic, caffeinated, or energetic drinks. Other ergogenic or stimulants were not controlled. The strength variables were measured in the same previous conditions on the second day. A schematic representation of the study design can be found in Figure 1.

#### 2.1.2. Measures

Bioimpedance analysis was used to assess body composition, i.e. body mass, body fat mass, lean mass, and fat-free mass, using a multifrequency tetrapolar bioimpedance previously validated device (MC780MA, Tanita Corp., USA) and a standard protocol of hydration and nutrition [14]. The volunteer wore light clothing and assumed a posture following the manufacturers’ instructions. The manufacturer’s software estimated segmental fat and lean mass for the trunk, arms, and legs (Biological Suite 8, Tanita).

For basal metabolism, MFO, and VO_2_max assessment, a mask was placed over the subject’s face to collect gas samples. Indirect calorimetry was measured using an open-circuit gas analyzer, Jaeger MasterScreen CPX® (CareFusion, San Diego, USA). Calibrations on the gas analyzer were performed daily before each assessment. During the test, the values of the gas analyzer were captured breath by breath and averaged every 20 s. Heart rate was measured continuously over the test with Polar Team 2 (Polar Electro Oy, Kempele, Finland). Basal metabolism was registered lying on a supine position for 30 min, and respiratory exchange ratio (RER) and fat oxidation calculations were based on respiratory VO_2_ and VCO_2_. A stable 5-min period with a coefficient of variation for VO_2_ and VCO_2_ below 15% was used to estimate substrate oxidation. All the exercise tests were performed on a cycle ergometer (Lode Excalibur, Groningen, The Netherlands) regulated in each volunteer’s seat and handlebar height. An incremental exercise protocol with two consecutive phases was designed to determine MFO, Fatmax, and VO_2_max. The first phase aimed to determine MFO, and it consists of several 3 min steps of 15W or 30W increments for low (<30 Kg/m^2^) and high (>30 Kg/m^2^) BMI, respectively, according to previously employed protocols to ensure an appropriate watts-per-kilogram ratio during the test [15], maintaining a pedaling cadence of 60–80 revolution per minute. The test was stopped upon reaching RER ≥ 1 to ensure the validity of the equation used for substrate calculations. The test usually takes from 6 to 28 min. After a short break (5 min), the second phase to detect the VO_2_max was initiated at the intensity that ended the last load of the previous stage and continued with steps of 15W per minute, with the same pedaling cadence. This phase ended when the participant reached voluntary exhaustion. This method of determining VO2max was validated by our laboratory with no significant differences from a full VO_2_max test. The criteria for determining VO_2_max include the detection of a VO_2_ plateau, reaching the age-predicted maximal heart rate ± 11 beats per minute, and meeting the respiratory exchange ratio (RER) criteria >1.15 and RPE > 18, as outlined by Wood et al. [16].

Fat oxidation was calculated during different steps of the protocol’s first phase, using the last registered minute of VO_2_ and VCO_2_ in each step and applying the equation proposed by Frayn [17]. Then, a parable was represented for each participant, showing the fat oxidation (g/min) at basal and different exercise intensities expressed as % of VO_2_max [8]. The MFO data was calculated from the curve using a third-degree polynomial iteration through the least squares method. The resulting equation was then used to determine the apex (MFO) and the corresponding intensity (Fatmax).

Two tests were used for the muscular strength assessment. A handgrip grip dynamometer with an adjustable grip (TKK 5101 Grip D; Takey, Tokyo, Japan) was used for the upper extremity strength assessment. Participants were asked to squeeze the dynamometer as hard as possible for at least 2 s, maintaining a standing anatomic position and with the elbow fully extended during the test. The device was adjusted for the volunteer grip span. The test registered the best of two attempts with each hand, and the mean of the best performance with each hand was retained for analyses. [18]

The standing longitudinal jump assessed lower extremity strength and power [19]. A week before the test, they were practised by the volunteers 3 to 6 times for correct execution. The long jump test was performed with separated feet at the width of the shoulders and with no restriction of arm movement. Then, participants were asked to jump with both feet as long as possible without falling. The distance from the standing position (start line) to the heel of the most backward foot at the final position was registered. The best of two attempts in centimeters was used for analyses.

### 2.2. Data analysis

The distribution of the variables was analyzed using Kolmogorov-Smirnov, and parametric analyses were made according to distribution. The independent *t-*test was used to analyze differences between males and females in the variables of physical characteristics. Data are presented as means ± standard deviation (SD).

A muscle strength score was computed, including handgrip and long jump variables. These variables were standardized as follows: standardized value = (value-mean)/SD, considering the sex and age from the values of handgrip [20] and long jump [21] tests. Then, the mean value between handgrip and long jump z-score was considered the strength cluster. Also, the cluster was relativized by lean mass. The Strength Cluster Extremity lean mass was calculated by handgrip/arm lean mass and long jump/leg lean mass. Then, simple and multiple linear regression models were performed to analyze the associations between body composition, VO_2_max, and muscle strength variables as independent variables and MFO and Fatmax as dependent variables. First, models were composed of the variables of interest in absolute values, then normalized by lean mass, and finally by the corresponding extremity lean mass, arms lean mass for handgrip, and leg lean mass for the other variables. Model 1 is unadjusted, absolute VO_2_max adjusts model 2, and model 3 is adjusted by total lean mass. The level of significance was set at *p* < 0.05.

Then, in the significant association variables, mediation analyses were performed to assess the possible role of absolute VO_2_max as a mediator in the association of handgrip, long jump, and strength cluster with MFO, according to Baron and Kenny criteria [22] with the PROCESS 4.1 macro for SPSS [23] using a bootstrap of ten thousand. The regression coefficients for three equations were calculated. In Equation 1, the total effect was calculated from the regression coefficient of the independent variable on the dependent variable (c). Equation 2 regressed the mediator (b) and the independent variable (c’) on the dependent variable. Equation 3 calculated the regression coefficient of the independent variable (a) on the mediator. To establish mediation, coefficients a, b, and c must be significant, and c’ must be attenuated. The indirect effect was calculated as the regression coefficient product of the mediator on the dependent variable (a) and the regression coefficient of the independent variable on the mediator (b). Finally, we calculated the Sobel Test to determine that the mediation effect was statistically significant.

## 3. Results

The descriptive characteristics of the overall sample segmented by sex are reported in Table 1.

**Table 1.** Descriptive characteristic of the overall sample by sex.

	Total (*n* = 81)	Men (*n* = 50)	Women (*n* = 31)	p
**Physical Characteristics**				
Age(years)	22.75 ± 4.42	22.26 ± 3.52	23.55 ± 5.55	.205
Height(cm)	171.76 ± 8.70	176.30 ± 6.42	164.44 ± 6.69	<.001
Weight(kg)	75.70 ± 16.04	78.34 ± 14.61	71.45 ± 17.52	.060
Body mass index (kg/m^2^)	25.70 ± 5.75	25.12 ± 4.16	26.64 ± 7.63	.251
Fat mass (kg)	18.66 ± 11.32	15.86 ± 9.09	23.17 ± 13.14	.009
Fat-free Mass (kg)	57.05 ± 9.21	62.48 ± 6.64	48.28 ± 5.08	<.001
Lean mass(kg)	54.19 ± 8.78	59.37 ± 6.34	45.84 ± 4.83	<.001
Arms lean mass (kg)	2.99 ± 0.76	3.49 ± 0.46	2.18 ± 0.30	<.001
Legs lean mass (kg)	18.82 ± 3.75	21.20 ± 2.51	14.98 ± 1.60	<.001
**Muscular strength**				
Handgrip (kg)	37.76 ± 9.17	43.31 ± 6.29	28.38 ± 4.29	<.001
Handgrip/lean mass (kg/kg)	0.69 ± 0.11	0.73 ± 0.10	0.63 ± 0.10	<.001
Handgrip/arm lean mass (kg/kg)	12.83 ± 2.02	12.53 ± 1.84	13.34 ± 2.25	.088
Long jump (cm)	184.97 ± 40.57	204.71 ± 22.58	153.40 ± 43.21	<.001
Long jump/lean mass (cm/kg)	3.48 ± 0.84	3.49 ± 0.57	3.45 ± 1.16	.821
Long jump/legs lean mass (cm/kg)	10.10 ± 2.61	9.81 ± 1.77	10.57 ± 3.55	.213
**Cardiorespiratory Fitness**				
VO_2_max (L/min)	3.03 ± 0.75	3.50 ± 0.51	2.28 ± 0.40	<.001
VO_2_max/kg (ml/kg/min)	41.22 ± 11.69	46.03 ± 9.99	33.35 ± 9.96	<.001
VO_2_max/kg lean mass (ml/kg/min)	56.07 ± 11.47	59.79 ± 10.58	50.01 ± 10.36	<.001
VO_2_max/kg leg lean mass (ml/kg/min)	162.33 ± 32.05	167.90 ± 30.91	153.22 ± 32.31	.048
**Fat oxidation**				
MFO (g/min)	0.37 ± 0.15	0.40 ± 0.16	0.32 ± 0.11	.019
MFO/body mass (g/min)	5.13 ± 2.23	5.36 ± 2.37	4.74 ± 1.96	.206
MFO/lean Mass (mg/min)	6.96 ± 2.75	6.91 ± 2.93	7.05 ± 2.50	.833
MFO/fat-free mass (g/min)	6.62 ± 2.61	6.56 ± 2.78	6.69 ± 2.37	.839
MFO/legs lean mass (g/min)	20.21 ± 7.97	19.38 ± 8.13	21.59 ± 7.64	.234
Fatmax (%VO_2_max)	40.98 ± 7.16	39.63 ± 7.79	43.17 ± 5.43	.032

Values are presented as mean ± standard deviation. T-test statistics were applied, and statistically significant differences between sexes are highlighted in bold. MFO: Maximal fat oxidation, VO_2_max: Maximal oxygen uptake.

Regression models were used to examine the relationships between lean mass, muscular strength, and VO_2_max with MFO in the overall sample. In the analysis of absolute variable values, the unadjusted linear regression model (model 1) revealed a direct association between long jump and VO_2_max with absolute MFO (*p* < 0.01). At the same time, handgrip and lean mass did not show significant associations. Introducing VO_2_max into the model (model 2) resulted in significant negative coefficients for handgrip, the strength cluster, lean mass, and leg lean mass, but no longer for the long jump test (Table 2). Notably, in model 3 introducing lean mass, only the long jump remained significantly associated with MFO (β standard = 0.322, *p* = 0.006).

**Table 2.** Associations of muscular strength, lean mass, and cardiorespiratory fitness with maximal fat oxidation in the overall sample.

	Model 1				Model 2			
*MFO*	*r*^*2*^	*β standard*	*95% CI*	*p*	*r*^*2*^	*β standard*	*95% CI*	*p*
Handgrip	0.04	0.192	−0.001;0.007	0.097	0.42	−0.331	−0090;-0.002	**0.006**
Long jump	0.11	0.328	0.000;0.002	**0.004**	0.39	−0.176	−0.002;0.000	0.164
Strength cluster	0.02	0.150	−0.017;0.080	0.199	0.36	−0.398	−0.118;-0.0216	**0.005**
Lean mass	0.01	0.094	−0.002;0.006	0.409	0.45	−0.359	−0.010;-0.003	**0.001**
Leg lean mass	0.02	0.150	−0.003;0.015	0.188	0.45	−0.369	−0.024;-0.006	**0.001**
*MFO/lean mass*								
*Handgrip/lean mass*	0.03	0.174	−0.001;0.010	0.133	0.43	−0.121	−0.008;0.002	0.216
*Long jump/lean mass*	0.14	0.271	0.001;0.002	**0.001**	0.43	−0.055	−0.001;0.001	0.624
*Strength cluster/lean mass*	0.14	0.371	0.001;0.002	**0.001**	0.42	0.036	−0.001;0.001	0.739
*MFO/leg lean mass*								
*Handgrip/arm lean mass*	0.13	0.360	0.001;0.002	**0.001**	0.44	0.111	0.000;0.001	0.247
*Long jump/leg lean mass*	0.15	0.386	0.001;0.002	**0.001**	0.45	−0.059	−0.001;0.001	0.602
*Strength cluster/extremities lean mass*	0.17	0.414	0.002;0.006	**<0.001**	0.43	−0.024	−0.002;0.002	0.842

MFO maximal fat oxidation, Model 1 unadjusted, Model 2 adjusted by VO_2_max (l/min). Statistically significant values are highlighted in bold.

Upon normalization by lean mass, model 1 showed significant associations between long jump/lean mass and strength cluster/lean mass with MFO/lean mass (*p* < 0.01). However, these associations lost significance after adjusting for VO_2_max/lean mass in model 2. Normalizing variables by leg lean mass in the unadjusted linear regression model 1 revealed significant associations of handgrip, long jump, and the strength cluster with MFO normalized by leg lean mass (*p* < 0.01). These associations ceased to be significant after adjusting for VO_2_max/leg lean mass.

Table 3 displays muscular strength, lean mass, and VO2max associations with Fatmax. In the unadjusted linear regression model (model 1) for absolute variables, the handgrip test and lean mass were inversely associated with Fatmax (*p* < 0.05). After adjusting for VO_2_max in model 2, the inverse associations between handgrip and Fatmax (*p* < 0.001) persisted, and lean mass was positively associated with Fatmax (*p* < 0.001). Model 3, adjusted for lean mass, revealed an association between the long jump test and Fatmax (*p* < 0.01).

**Table 3.** Associations of muscular strength, lean mass and cardiorespiratory fitness with fatmax in the overall sample.

	Model 1				Model 2			
*Fatmax*	*r*^*2*^	*β standard*	*95% CI*	*p*	*r*^*2*^	*β standard*	*95% CI*	*p*
Handgrip	0.08	−0.284	−0.409;-0.050	**0.013**	0.21	−0.591	−0.695;-0.259	**<0.001**
Long jump	0.00	0.000	−0.041;0.041	0.998	0.02	−0.114	−0.076;0.036	0.479
Strength cluster	0.01	0.078	−1.567;3.151	0.506	0.01	0.063	−1.776;3.049	0.600
Lean mass	0.08	−0.289	−0.426;-0.060	**0.010**	0.17	−0.493	−0.626;-0.203	**<0.001**
Leg lean mass	0.07	−0.270	−0.954;-0.100	**0.020**	0.18	−0.526	−1.546;-0.511	**<0.001**
*Handgrip/lean mass*	0.12	−0.118	−22.986;7.363	0.308	0.17	−0.301	−35.348;-4.413	**0.012**
*Long jump/lean mass*	0.03	0.160	−0.587;3.306	0.168	0.08	−0.027	−2.643;2.186	0.851
*Strength cluster/lean mass*	0.02	0.121	−0.976;3.112	0.301	0.09	−0.054	−2.818;1.864	0.686
*Handgrip/arm lean mass*	0.01	0.082	−0.534;1.118	0.483	0.13	−0.072	−1.109;0.596	0.550
*Long jump/leg lean mass*	0.03	0.180	−0.132;0.142	0.120	0.11	−0.049	−0.916;0.648	0.734
*Strength cluster/extremities lean mass*	0.02	0.122	−0.927;2.982	0.298	0.14	−0.177	−3.925;0.942	0.226

Model 1 unadjusted, Model 2 adjusted by VO_2_max (l/min).

When variables were normalized by lean mass, model 1 showed that VO_2_max normalized by lean mass was associated with Fatmax (*p* = 0.01), with no significant associations for other variables. After adjusting for VO_2_max/lean mass (model 2), no associations of muscular strength with Fatmax were observed. Normalizing variables by leg lean mass in the unadjusted linear regression model 1 indicated that VO_2_max normalized by leg lean mass was associated with Fatmax (*p* = 0.003). Still, after adjusting for VO_2_max/leg lean mass (model 2), no associations with muscular strength variables were found.

All MFO and Fatmax models were adjusted for age and sex as covariates, and the results remained consistent.

**Figure 2.**
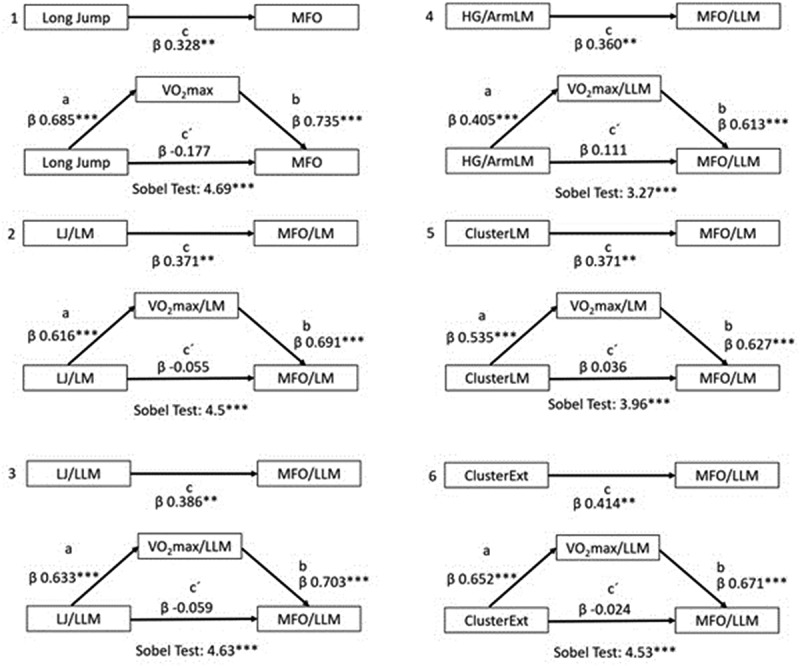
Mediation model for the association of strength variables and maximal fat oxidation with the mediation of cardiorespiratory fitness. a: independent variable (IV) effect on the mediator (M); b: M effect on the dependent variable (DV); c: IV effect on DV; c:’ direct effect; LJ: longitudinal jump; LM: lean mass; LLM: leg lean mass; MFO: maximal fat oxidation; VO2max: maximum oxygen uptake; Ext: Extremities; cluster: mean standardized z score of handgrip and longitudinal jump. 1) IV: LJ, M: VO2max, DV: MFO; 2) IV: LJ/LM, M: VO2max/lm, DV: MFO/LM; 3) IV: LJ/LLM, M: VO2max/llm, DV: MFO/LLM; 4) IV: HG/ArmLM, M: VO2max/llm, DV: MFO/LLM; 5) IV: SC/LM, M: VO2max/lm, DP: MFO/LM; 6) IV: cluster Ext, M: VO2max/llm, DP: MFO/LLM. ***: *p* < 0.001; **p < 0.01. β: standardized regression coefficient.

Mediation analysis between muscular strength, VO_2_max, and MFO indicated that six variables met the Baron and Kenny criteria. These groups were analyzed for mediation; the results are depicted in Figure 2. Notably, long jump showed standardized indirect effects on MFO, mediated by VO_2_max (0.504; CI: 0.319–0.697); normalizing by lean mass (0.426; CI: 0.265–0.602), and normalizing by leg lean mass (0.445; CI: 0.296–0.617), confirmed by the Sobel test (all *p* < 0.01). The handgrip/arm lean mass showed indirect effect on MFO/leg lean mass mediated by VO_2_max/leg lean mass (0.248; CI:0.119–0.378). The cluster variables also exhibited mediation of MFO by VO_2_max/lean mass (0.336; CI: 0.204–0.479) and VO_2_max/legs lean mass (0.437; CI: 0.284–0.601) (*p* < 0.01).

## 4. Discussion

The main finding of this study was that MFO is indirectly influenced by muscle strength. The analysis of the simple and multiple regression models suggests a mediation role of VO_2_max in the relationship between muscular strength, muscle quality and MFO as an indirect effect. The participants showed differences in body composition, VO_2_max, muscular strength, and MFO between men and women. Normalizing the muscular strength, VO_2_max, and MFO variables by the lean and leg lean mass can adjust these differences and clarify the role of the metabolically active lean mass in the MFO. Literature has shown that body composition variables like body mass, BMI, fat mass, and lean mass are associated with MFO [7,8]. The muscle is the main metabolically active tissue where exercise oxidative metabolism occurs, and its quantity has been identified as a determinant of MFO and Fatmax [24]. Our results found significant inverse associations of lean mass with MFO when adjusted by VO_2_max, but not in an unadjusted model. Contrarily, a direct relationship exists between MFO and lean mass in an obese and older population [10]. Interestingly, studies considering different BMI categories have shown that overweight and obese adults with higher levels of lean mass than their normal-weight counterparts had lower fat oxidation when normalized by lean mass [25].

The phenomenon of variation in muscle mass oxidative capacity concerning exercise intensity is known as metabolic flexibility [26]. This concept holds significant clinical implications, particularly for overweight and obese individuals who exhibit a limited capacity to oxidize fat during exercise per unit of metabolically active tissue, possibly due to lower levels of VO_2_max. To improve the analysis, we normalized MFO by total lean mass and extremities lean mass to understand MFO per unit of metabolically active tissue or metabolic muscle quality. We also have normalized muscular strength by lean mass and extremities lean mass to obtain muscle quality or functional, active tissue. The adjusted variables can clarify the relationship between muscular characteristics and fat oxidation, which has been barely explored in the literature.

Our results have shown a significant association of strength with MFO, normalized by total and extremities lean mass. An enhanced muscle quality [13] can be related to better metabolic response rather than considering only total strength, and the association with the metabolic muscle quality (MFO/lean mass and MFO/legs lean mass) can be an index of healthy muscular function, more reliable than absolute MFO. It is well known that the size of the high oxidative type I fibers is smaller compared to the stronger type II fibers, this can affect the total size and, finally, the cross-sectional area, regarding the consideration of the total size of the muscle can mask the capability for fat oxidation [27]. This observation suggests that muscle quality and not only quantity are important in maximal fat oxidation.

Various types of muscle fibers can account for the variations in fat oxidation between lean individuals and those who are obese. It is reported in the literature [28] that persons with increased body mass have more type II and fewer type I muscle fibers. Additionally, individuals with strength-trained musculature characterized by a significant proportion of type II muscle fibers, which possess high force-generation capabilities but lower metabolic capacity [27], this difference is particularly noticeable. Also, other factors related to fat availability, transport, and hormone sensitivity within the muscle can influence fat oxidation during exercise [29]. In high body mass persons, the fat deposits are more central, which can produce alterations in metabolism in these muscle fibers [30].

Regarding Fatmax, obese adults reported values of 43.3 ± 13.5% of VO_2_max on a cycle ergometer [12], similar to our sample (41.0% ±7.2). Fatmax has an inverse association with handgrip, even when adjusted by VO_2_max. However, considering the Fatmax regression models normalized by lean mass, the association is no longer significant for the strength variables but remains significant for VO_2_max. The normalization models by lean mass suggest that muscle strength is not an important factor in Fatmax. Nevertheless, VO_2_max normalized by total, and leg lean mass was directly associated with Fatmax, similar to previous studies [7,31]. The metabolic capability of producing high energy levels from fat oxidation is related to VO_2_max determinants [32], explaining this significative association in all normalized models.

The mediation analysis calculates the indirect effect based on the product of the coefficient of muscular strength/quality variables on VO_2_max (a) and the coefficient of VO_2_max on MFO (b). The mediation role of VO_2_max on muscular strength and quality variables in the association with MFO was significant by the Sobel test (Figure 2). To establish mediation analysis, there are four essential criteria [23], one of which involves comparing the total effect (c) reduction with the coefficient c.’ Furthermore, in our study, there is a change in sign between c and c,’ which indicates inconsistent mediation [33]. Inconsistent mediation suggests that VO_2_max acts as a suppressed factor in the relationship, with muscular strength relatively low in comparison.

In our findings, the indirect effect of the long jump on MFO is mediated by VO_2_max, both in absolute terms and when normalized by lean and leg lean mass. The assessment of the long jump directly involves evaluating lower limb lean mass, which indicates muscle quality. This muscle quality is associated with the aerobic metabolic capacity expressed in MFO. Similarly, the handgrip indirect effect on MFO is mediated by VO_2_max, but only when normalized by arm lean mass. Although arm lean mass is not directly involved in determining MFO, handgrip is a reliable indicator of muscle mass and strength in adults. This can explain why this mediation is considered an indicator of muscle quality. In the case of the strength cluster, normalization by lean mass and extremities lean mass aligns with the mediation of VO_2_max on MFO. In all these cases, the coefficient effects increase when functionally active lean mass is considered due to normalizing the strength variables by total lean mass and extremities lean mass.

This phenomenon can be explained by the fact that the quality of the muscles involved in the test is relevant to an individual’s MFO. In contrast, the overall body lean mass is not necessarily linked to the metabolic capability of fat oxidation through exercise. It is important to differentiate the capacities of the skeletal muscle and consider the muscle unit to analyze them. Strength capacity is associated with quality of life and independence, but high muscle strength can have limitations regarding metabolic capacity. Further studies are required to confirm the hypothesis that metabolic and functionally active lean mass plays a significant role in MFO.

This study has some limitations that should be considered. The cross-sectional design does not allow us to determine causal links between variables. Regarding the muscular strength assessment, we used the handgrip strength test for the upper limb, which is highly correlated with health parameters [34], and the long jump test for the lower limb [35]. Both tests are reliable and cheap for non-clinical applications [36], although ideally an isokinetic dynamometer should have been used to assess lower body strength. Therefore, laboratory-based tests must be used in the future to determine muscular strength and its relationship with fat oxidation. In addition, our sample comprised young adults without diagnoses of metabolic disease, so the extrapolation of results should consider this. Future studies need to include a larger sample with more significant body composition heterogeneity to understand better the role of MFO per unit of active tissue. We also did not control the participant's diet, a variable that directly influences MFO, which is another limitation of our study. Finally, body composition was measured by bioimpedance instead of using another more precise method like Dual Energy X-ray Absorptiometry (DXA).

Finally, our data show that muscular strength has a role not only in VO_2_max but also in MFO. This observation can be an argument to include the evaluation of strength in an attempt to identify those at higher risk of those pathologies associated with reduced fat oxidation capacity. Our finding suggests that the quality of the muscles and not only the quantity are important as determinants of MFO. However, further studies are needed to understand the relationship between strength, VO_2_max, and MFO with larger samples and the effect of strength training using a randomized control trial design.

## 5. Conclusion

In conclusion, our study highlights that muscular strength and quality indirectly impact MFO, mediated by VO_2_max. Additionally, it emphasizes the significance of muscle quality, considering muscles as metabolically active and functional tissue, which adds further importance to health and performance. These findings shed light on the interplay between muscle quality and CRF with MFO, contributing to a better understanding of the factors influencing metabolic capabilities.

## 6. Practical implications

- Maximal fat oxidation is influenced by cardiorespiratory fitness, lean mass, and muscle strength.
- To analyze fat oxidation determinants, it is necessary to normalize them using lean mass.
- The prescription of exercise with metabolic objectives needs to consider strength training focused on quality rather than lean mass.

## Data sharing statement

The identified data is available from the corresponding author upon reasonable request.
